# Hepatocellular carcinoma in elderly: Clinical characteristics, treatments and outcomes compared with younger adults

**DOI:** 10.1371/journal.pone.0184160

**Published:** 2017-09-08

**Authors:** Hui Guo, Tao Wu, Qiang Lu, Jian Dong, Yi-Fan Ren, Ke-Jun Nan, Yi Lv, Xu-Feng Zhang

**Affiliations:** 1 Department of Hepatobiliary Surgery, the First Affiliated Hospital of Xi’an Jiaotong University, Xi’an, China; 2 Department of Oncology, the First Affiliated Hospital of Xi’an Jiaotong University, Xi’an, China; 3 Institute of Advanced Surgical Technology and Engineering, the First Affiliated Hospital of Xi’an Jiaotong University, Xi’an, Shaanxi Province, China; University of North Carolina at Chapel Hill School of Medicine, UNITED STATES

## Abstract

The number of elderly patients diagnosed with hepatocellular carcinoma (HCC) is expected to increase. The present study aims to evaluate the role of age on treatments and outcome of HCC patients. 1530 patients firstly diagnosed with HCC were retrospectively included and classified as older (≥65 years, n = 318, 21%) and younger patients (<65 years, n = 1212, 79%). The two groups were compared with clinical characteristics, tumor burden, Barcelona Clinics Liver Cancer (BCLC) stage, treatments and long-term prognosis. Elderly patients were more HCV infected, had more diabetes, poorer performance status, and were less aggressively treated. The proportion of HCC within BCLC stage 0-A, B or C was similar between the two groups, but elderly patients were more presented with BCLC stage D. The overall survival of older patients was poorer compared to younger patients before and after propensity score matching. However, elderly patients were less often effectively treated with surgery and loco-regional therapies across different BCLC stages. After stratified by BCLC stages or treatments, older patients showed comparable long-term outcome to younger patients. Performance status, BCLC stages and effective treatments, rather than age, was independent factors determining prognosis in the whole cohort and only elderly patients by multivariate analysis. In conclusion, older could have comparable survival to younger patients within the same tumor stage or after similar treatments. Thus, equally active treatments should be encouraged to elderly patients.

## Introduction

The aging of the population is a global phenomenon. The number of elderly adults with different types of malignant tumors has been increasing with longer life expectancy of the population. It is expected that 60% of all cancers will be detected in elderly patients, and treatment of elderly cancer patients remains a challenge worldwide [1]. However, aging is associated with a progressive reduction in the functional reserve of multiple organ systems, increased disability rates and reduced tolerance of physical, emotional, and social stress [1, 2]. Therefore, it was found that old age was associated with poorer prognosis in prostate cancer [3], thyroid cancer [4], lung cancer [5], etc. However, in some other cancers including colorectal [6], breast [7], and gastric cancers [8], older patients had improved outcome than younger patients. Therefore, aging might have distinct impacts on outcome of patients with different types of cancers.

Age affects liver less than the musculoskeletal and cardiovascular systems [9, 10]. Hepatocellular carcinoma (HCC) is the second most common cause of cancer mortality worldwide [11, 12]. Several well-known factors such as tumor size, number, macrovascular invasion, etc. are associated with prognosis of HCC patients [13]. Of note, age has been reported to play an important role in HCC outcome. Nevertheless, studies on the clinical features, therapeutic options, and survival of older adults with HCC have been limited and debatable, despite an increasing clinical need to manage older adults with HCC [10, 14–17]. The discrepancy between the different studies may be due to the diverse demographic characteristics, clinicopathologic features, and treatment modalities available to patients.

The chronological age of 65 is currently accepted as a threshold to define an “elderly” person socially. Therefore, we conducted the present study in an HBV endemic area to determine HCC characteristics, treatment modalities, safety, and prognosis in older patients (≥65 years) in comparison with synchronous younger counterparts.

## Patients and methods

### Patient enrollment

From 2008 to 2013, 1530 adult patients (≥ 18 years) firstly diagnosed with HCC in the largest tertiary hospital in Northwest China were included with intact clinical information and continuous follow-up. The diagnosis of HCC was made by computed tomography scan, ultrasonography, magnetic resonance image and/or angiography preoperatively, and confirmed by histopathological examination of the resected specimen postoperatively if available. The patients were divided into two groups according to the age at admission: the younger (<65 years) and older group (≥65 years).

Tumor status was evaluated by imaging studies, and pathological examination if available. Barcelona Clinic Liver Cancer (BCLC) system was used as the optimal staging system in the present study. Since tumor size is a critical factor determining treatment outcome, we and others recommended single tumor larger than 5 cm should be classified as BCLC stage B [18, 19]. Surgical resection was the first-line treatment for patients with BCLC stage A and B after evaluation of tumor size, liver function, remnant liver volume, patient general condition and willingness. Patients with unresectable tumor or unwillingness of surgical treatment would receive loco-regional treatments (LRT), including palliative radiofrequency ablation (RFA), transarterial chemoembolization (TACE), and percutaneous ethanol injection (PEI). However, RFA treatment of HCC within BCLC stage 0-A was recognized as curative treatment and therefore separated from LRT group. Those refusing any invasive treatments might be treated with best supportive treatment (BST). This study has been approved by the ethics committee of Xi’an Jiaotong University and The First Affiliated Hospital. A waiver of informed consent was obtained, since the data were analyzed from the electronic medical record and reported without personal identifiers.

### Statistical analysis

Numerical data was expressed as median and range, and compared by Mann-Whitney U test or *t* tests, whereas nominal variables were expressed as number and percentages and compared by Chi-Square test or Fisher’s exact test. The overall survival rates were calculated by the Kaplan-Meier method, and the differences in survival between groups were compared using the log-rank test. Multivariate analysis was performed and hazard ratio (HR) and 95% coincidence interval (CI) were calculated with forward stepwise Cox proportional hazard regression analysis. Statistical analysis was carried out using SPSS 22.0. *p*<0.05 was considered statistically significant.

To eliminate the selection bias, we introduced PSM analysis into the present study to balance the baseline differences of the groups and thereby simulate random group allocation [20]. 1:1 matching without replacement was performed using a caliper with a width 0.01 of the standard deviation to generate matched pairs of the patients.

## Results

### General characteristics

318 (20.8%) patients were above 65 years old (older group), while 1212 (79.2%) were younger patients. The general characteristics of the patients in the two groups were compared (Table 1). More older patients had diabetes, high Eastern Cooperative Oncology Group (ECOG) score, HCV infection and were unlikely to be routinely screened for HCC previously, in comparison with younger (all *p*<0.01). In contrast, more young patients were cigarette smokers and alcohol abusers, and had HBV infection and liver cirrhosis (all *p*<0.05).

**Table 1:** Demographic, clinical and tumor characteristics, and treatments of young and elderly patients with HCC.

Variables	Young (<65 y) (n = 1212)	Elderly (≥65 y) (n = 318)	P value
Gender (male/female)	988/224	250/68	0.262
Child-Pugh class			0.048
A	1072 (88.4%)	265 (83.3%)	
B	126 (10.4%)	47 (14.8%)	
C	14 (1.2%)	6 (1.9%)	
Smoking history	535 (44.1%)	120 (37.7%)	0.040
Alcohol abuse	310 (25.6%)	60 (18.9%)	0.012
Diabetes	95 (7.8%)	43 (13.5%)	0.003
ECOG score			<0.001
0	962 (79.4%)	209 (65.7%)	
1	213 (17.6%)	68 (21.4%)	
≥2	37 (3.1%)	41 (12.9%)	
Hepatitis status			<0.001
Hepatitis B	966 (79.7%)	157 (49.4%)	
Hepatitis C	39 (3.2%)	48 (15.1%)	
Hepatitis B + C	5 (0.4%)	2 (0.6%)	
None	202 (16.7%)	111 (34.9%)	
Routine HCC screening	647 (53.4%)	139 (43.7%)	0.009
Liver cirrhosis	847 (69.9%)	168 (52.8%)	<0.001
First Dept. admitted			0.013
Surgery	999 (82.4%)	239 (75.2%)	
Internal Medical Dept.	191 (15.8%)	70 (22.0%)	
Other Dept.	22 (1.8%)	9 (2.8%)	
Tumor size (>5 cm)	707 (58.3%)	196 (61.6%)	0.306
Multinodular tumor	692 (57.1%)	153 (48.1%)	0.004
Macrovascular invasion	213 (17.6%)	48 (15.1%)	0.316
BCLC stages			<0.001
0-A	362 (29.9%)	86 (27.0%)	
B	603 (49.8%)	144 (45.3%)	
C	197 (16.3%)	43 (13.5%)	
D	50 (4.1%)	45 (14.2%)	
Primary treatments			<0.001
Liver transplant	34 (2.8%)	0	
Surgical resection	383 (31.6%)	78 (24.5%)	
Radiofrequency ablation†	86 (7.1%)	32 (10.1%)	
Loco-regional therapies	444 (36.6%)	101 (31.8%)	
Supportive treatments	265 (21.9%)	107 (33.6%)	
30-day mortality	59 (4.9%)	16 (5.0%)	0.884
90-day mortality	114 (9.4%)	42 (13.2%)	0.048
Median survival (months)	33 (0–108)	27 (0–104)	0.002

^†^Only for HCC within BCLC stage 0-A.

HCC, hepatocellular carcinoma; ECOG, the Eastern Cooperative Oncology Group; BCLC, Barcelona Liver Cancer; Dept., Department.

Interestingly, more young patients displayed multiple lesions than elderly (*p* = 0.004), although tumor size was not significantly different between them. Although similarly distributed in BCLC stages 0-A, B and C of HCC were the two groups, more elderly patients presented with end-staged HCC (BCLC D) at admission (*p*<0.01). Therefore, the older patients tended to be admitted within non-surgery departments for non-surgical treatments. As predicted, older patients were less frequently treated with surgery in comparison with younger patients (24.5% *vs*. 34.4%, *p*<0.001, Table 1). As a curative therapy with minimal invasiveness, radiofrequency ablation was marginally more performed in the selected younger than older patients with BCLC 0-A staged HCC (*p* = 0.078). More elderly patients received no liver-directed therapy but only supportive care (33.6% *vs*. 21.9%, *p*<0.001)

### Overall survival before and after propensity score matching

**Fig 1:**
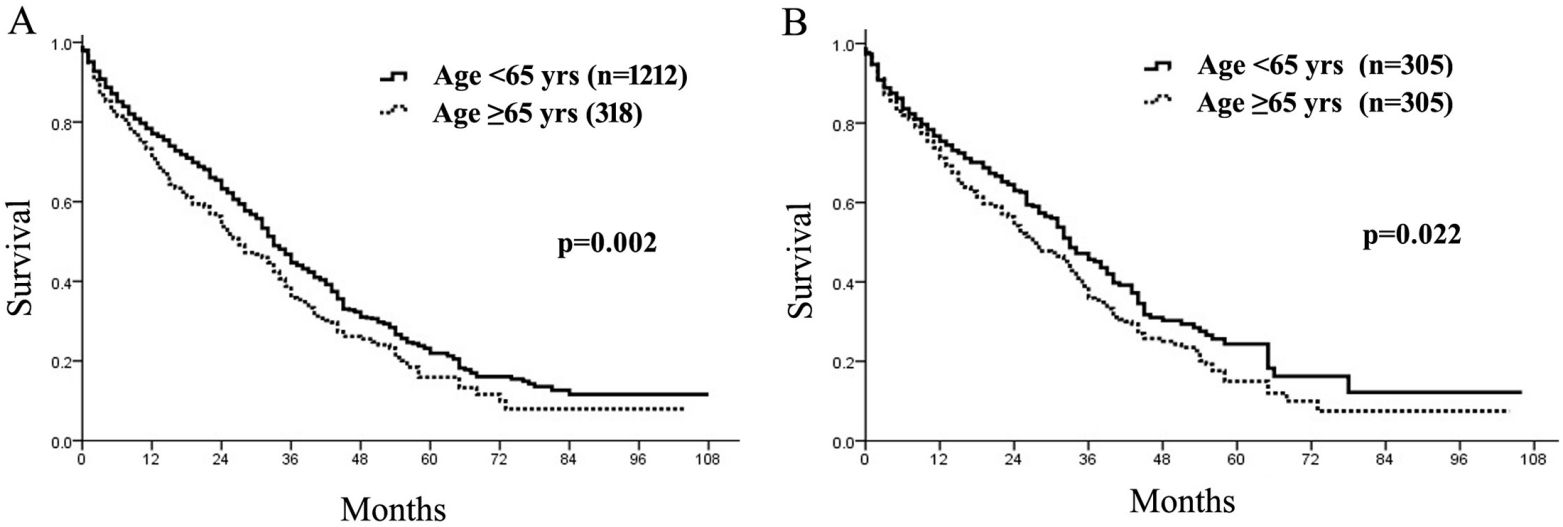
Overall survival of older and younger patients with hepatocellular carcinoma before (A) and after (B) propensity score matching.

Although the 30-day mortality between the two groups was similar (older, 5.0% vs. younger, 4.9%, *p* = 0.884), the 90-day mortality seemed to be higher in older than younger patients (older, 13.2% vs. younger, 9.4%, *p* = 0.048). The global median, 1-, 3-, and 5-year survival rates of older patients were 27 months, 71%, 36%, and 16%, which was significantly worse than 33 months, 77%, 44%, and 21% of younger patients (*p* = 0.002, Fig 1A). Given the base differences in older and younger cohorts, PSM was then utilized to generate 305 pairs of well-matched patients with similar preoperative performance status, liver function, tumor size, number, vascular invasion, and BCLC stages (Table 2). As demonstrated, older patients were less often to receive curative treatments including surgical resection, liver transplant and RFA (only for BCLC 0-A staged HCC) than younger (30.5% *vs*. 42%, *p* = 0.003, Table 2). The 30- and 90-day mortality were similar between older and younger groups (both *p*>0.05, Table 2). Therefore, it is not surprising that overall survival of older patients was poorer than their younger counterparts (median survival, 27 months *vs*. 33 months, *p* = 0.022, Fig 1B). These findings implied that even with similar host factors and tumor burden, older patients tended to be less aggressively treated than younger patients, which might account for the diminished long-term outcome of elderly patients.

**Table 2:** Demographic and clinical characteristics of the younger and elderly patients with HCC in propensity model.

Variables	Young (<65 y) (n = 305)	Elderly (≥65 y) (n = 305)	P value
Male gender	239 (78.4%)	239 (78.4%)	1.000
ECOG score ≥1	96 (31.5%)	96 (31.5%)	1.000
Child-Pugh Class B/C	46 (15.1%)	46 (15.1%)	1.000
Smoking history	127 (41.6%)	114 (37.4%)	0.282
Alcohol abuse	66 (21.6%)	57 (18.7%)	0.364
Diabetes	33 (10.8%)	41 (13.4%)	0.321
Liver cirrhosis	168 (55.1%)	168 (55.1%)	1.000
Tumor size (>5 cm)	180 (59.0%)	190 (62.3%)	0.407
Multinodular tumor	155 (50.8%)	141 (46.2%)	0.257
Macrovascular invasion	51 (16.7%)	46 (15.1%)	0.580
BCLC stages			1.000
0-A	82 (26.9%)	82 (26.9%)	
B	144 (47.2%)	144 (47.2%)	
C	43 (14.1%)	43 (14.1%)	
D	36 (11.8%)	36 (11.8%)	
Primary treatments			<0.001
Liver transplant	10 (3.3%)	0	
Surgical resection	88 (28.9%)	75 (24.6%)	
Radiofrequency ablation †	30 (9.8%)	18 (5.9%)	
Loco-regional therapies	117 (38.4%)	110 (36.1%)	
Supportive treatments	60 (19.7%)	102 (33.4%)	
30-day mortality	16 (5.2%)	16 (5.2%)	1.000
90-day mortality	34 (11.1%)	39 (12.8%)	0.533
Median survival (months)	33 (0–106)	27 (0–104)	0.022

^†^only for HCC within BCLC stage 0-A.

ECOG, the Eastern Cooperative Oncology Group; BCLC, Barcelona Liver Cancer; Dept., Department.

### Treatments and survival in different BCLC stages

**Fig 2:**
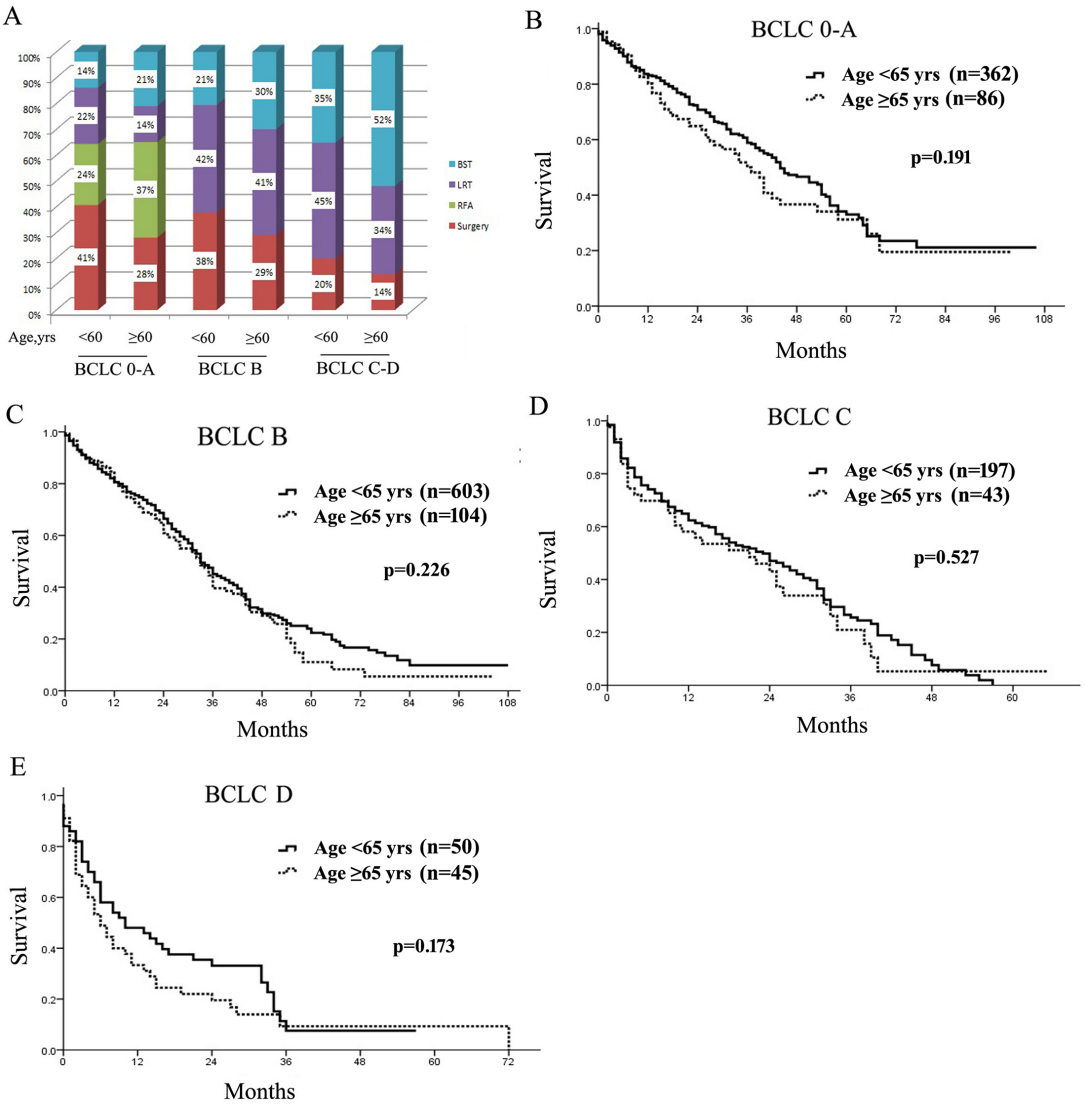
Treatments (A) and overall survival (B-E) of older and younger patients according to different Barcelona Clinics Liver Cancer (BCLC) stages of hepatocellular carcinoma. RFA, radiofrequency ablation; LRT, loco-regional therapies; BST, best supportive treatments.

Furthermore, we evaluated any difference of treatment modalities at different BCLC stages of HCC between older and younger patients including surgical treatments (resection and liver transplant), LRT and BST. Consistently, elderly individuals were less effectively treated by surgery or LRT but more by BST across different BCLC stages (Fig 2A). However, in BCLC stage 0-A, older patients were equally treated with curative therapies (surgical resection, RFA and liver transplant in total) (65% *vs*. 65%) to younger patients, although RFA was more frequently performed in older patients because of its minimal invasiveness (37% *vs*. 24%, Fig 2A).

Even though being less effectively treated, older patients showed no significant difference in long-term survival across different BCLC stages compared with younger (median survival, BCLC 0-A, 37 months *vs*. 44 months, BCLC B, 27 months *vs*. 30 months, BCLC C, 21 months *vs*. 23 months, and BCLC D, 6 months *vs*. 10 months, all *p*>0.05, Fig 2B, 2C, 2D and 2E).

### Survival after different treatments

**Fig 3:**
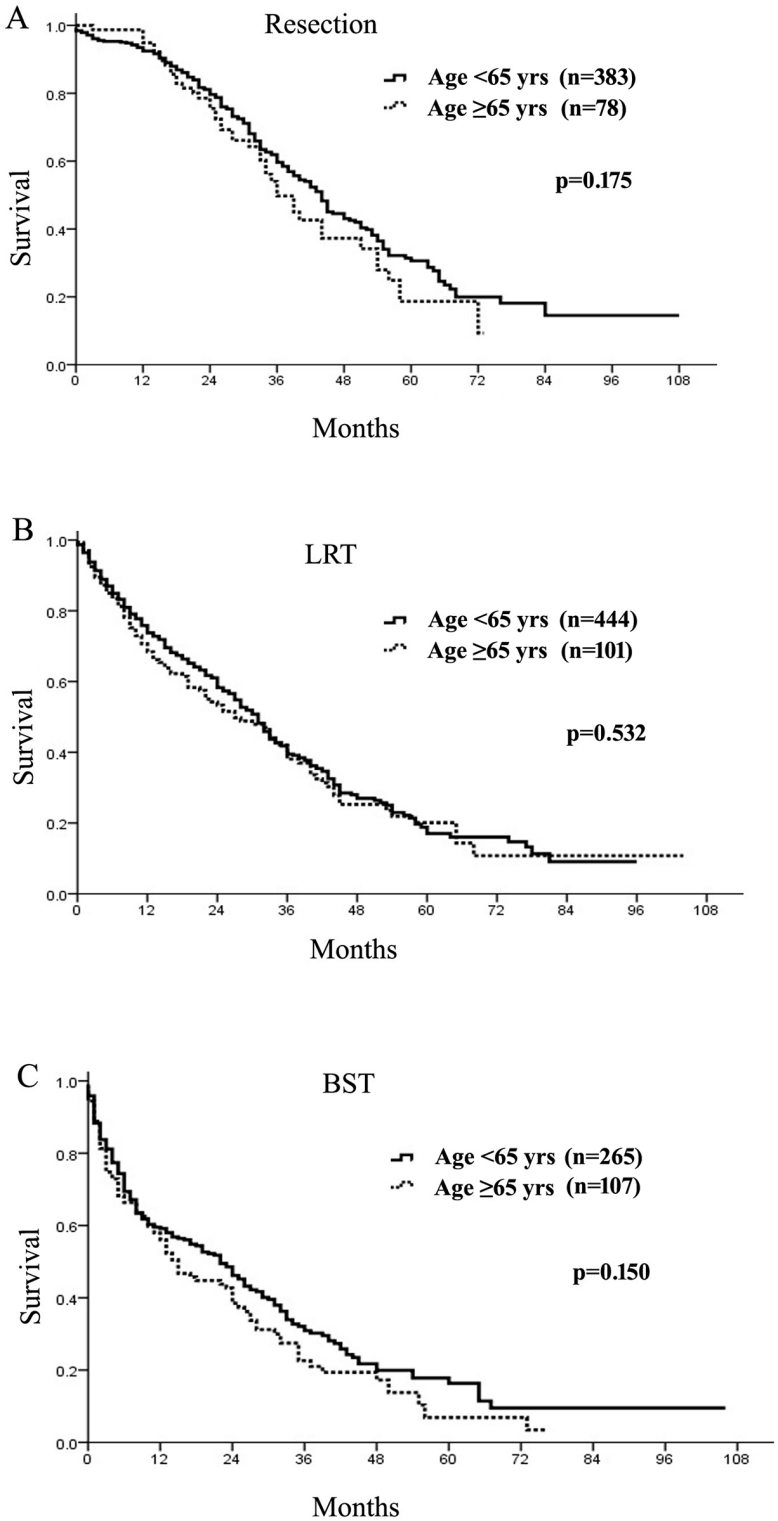
Overall survival of older and younger patients after surgical resection (A), loco-regional therapies (LRT, B) and best supportive treatments (BST, C).

Impressively, after similar treatments, overall survival of older patients was equivalent to younger ones after surgical resection, LRT or BST, respectively (median survival, 36 months *vs*. 44 months, 27 months *vs*. 31 months, and 15 months *vs*. 22 months, all *p*>0.05, Fig 3A, 3B and 3C).

### Factors affecting long-term survival

We evaluated the factors affecting long-term survival of all the HCC patients and only elderly patients by univariate and multivariate analysis (Tables 3 and 4). By multivariate analysis, age was not an independent risk factor affecting the prognosis of patients. Consistently, in the whole cohort and only elderly, high ECOG score and advanced BCLC stages were associated with poorer prognosis, while curative treatments and LRT versus BST were associated with prolonged survival (Tables 3 and 4).

**Table 3:** Analysis of factors affecting survival of 1530 HCC patients using proportional hazards regression model.

Factors	comparison	Univariate analysis		Multivariate analysis	
		HR (95% CI)	P value	HR (95% CI)	P value
Sex	Male vs. female	1.0 (0.8–1.2)	0.935		
Age (years)	≥65 vs. <65	1.3 (1.1–1.5)	0.002	1.1 (0.9–1.3)	0.281
Child-Pugh	B-C vs. A	1.7 (1.4–2.0)	<0.001	1.2 (0.9–1.4)	0.156
Smoking	Yes vs. no	1.1 (0.9–1.2)	0.374		
Alcohol	Yes vs. no	1.0 (0.9–1.2)	0.715		
Diabetes	Yes vs. no	0.9 (0.7–1.2)	0.448		
ECOG	≥1 vs. 0	1.6 (1.3–1.8)	<0.001	1.4 (1.2–1.6)	<0.001
Hepatitis	None	1			
	B	1.1 (0.8–1.5)	0.571		
	C	0.9 (0.7–1.2)	0.456		
Liver cirrhosis	Yes vs. no	1.1 (0.9–1.2)	0.243		
BCLC stage	0-A	1		1	
	B	1.3 (1.1–1.6)	<0.001	1.3 (1.1–1.5)	<0.001
	C-D	2.8 (2.4–3.4)	<0.001	2.4 (2.0–2.8)	<0.001
Treatments	BST	1			
	Curative treatments	0.4 (0.4–0.5)	<0.001	0.5 (0.4–0.6)	<0.001
	LRT	0.7 (0.6–0.8)	<0.001	0.8 (0.7–0.9)	<0.001

HCC, hepatocellular carcinoma; ECOG, the Eastern Cooperative Oncology Group; BCLC, Barcelona Clinic Liver Cancer; BST, best supportive treatment; LRT, loco-regional therapies.

**Table 4:** Analysis of factors affecting survival of elderly HCC patients (≥65 years) using proportional hazards regression model.

Factors	comparison	Univariate analysis		Multivariate analysis	
		HR (95% CI)	P value	HR (95% CI)	P value
Sex	Male vs. female	1.4 (1.0–2.0)	0.041	1.3 (0.9–1.0)	0.137
Child-Pugh	B-C vs. A	1.9 (1.4–2.7)	<0.001	1.1 (0.7–1.6)	0.688
Smoking	Yes vs. no	1.4 (1.1–1.8)	0.015	1.3 (1.0–1.7)	0.102
Alcohol	Yes vs. no	1.1 (0.8–1.6)	0.445		
Diabetes	Yes vs. no	1.1 (0.7–1.6)	0.622		
ECOG	≥1 vs. 0	2.1 (1.6–2.7)	<0.001	1.6 (1.2–2.2)	0.002
Hepatitis	None	1			
	B	1.4 (0.9–2.1)	0.108		
	C	1.0 (0.7–1.5)	0.946		
Liver cirrhosis	Yes vs. no	0.9 (0.7–1.2)	0.480		
BCLC stage	0-A	1		1	
	B	1.3 (0.9–1.8)	0.186	1.1 (0.8–1.6)	0.518
	C-D	3.0 (2.1–4.3)	<0.001	2.3 (1.6–3.4)	<0.001
Treatments	BST	1		1	
	Curative treatments	0.4 (0.3–0.6)	<0.001	0.5 (0.4–0.8)	0.001
	LRT	0.6 (0.5–0.9)	0.003	0.7 (0.5–1.0)	0.068

HCC, hepatocellular carcinoma; ECOG, the Eastern Cooperative Oncology Group; BCLC, Barcelona Clinic Liver Cancer; BST, best supportive treatment; LRT, loco-regional therapies.

## Discussion

In the present study, we initially observed a poorer long-term survival of elderly patients with HCC in comparison with young patients. In the propensity model with well match of the host- and tumor-related factors, older patients were found having been less often curatively treated, which possibly accounted for the poorer overall survival of them than younger patients. It was also consistently demonstrated that elderly patients were unlikely to be surgically (surgical resection and liver transplant) or effectively (surgery and LRT) treated in different BCLC stages. Therefore, we stratified the patients by HCC stages or treatments. And it was clearly shown that overall survival was not significantly different between older and younger patients within the same BCLC stages or after similar treatments. Age was not found an independent risk factor determining prognosis. ECOG score and tumor stages should be the most important factors considered rather than age for treatment selection in elderly patients. These findings were consistent with some other studies that surgical resection and some non-surgical treatments attained the equivalent long-term survival in older with younger patients [10, 14, 15, 21, 22] (Table 5). Therefore, older patients can have comparable long-term outcome to younger patients when appropriately selected and treated.

**Table 5:** Clinicopathological features and overall survival of younger and elderly patients from literatures.

Study	Period	Country/region	Age groups	n/n	Older group compared with younger group	Overall survival
Cho et al. [16]	1987–2003	Korea	<30, 30–60, ≥60	71/168/81	More cirrhotic, earlier disease stage	Better (*p* = 0.007)
Mirici-Cappa et al. [26]	1987–2004	Italy	<70, ≥70	1104/614	More comorbidities, better liver function, higher CLIP score, more percutaneous but less surgical or TACE treatment	Similar (*p* = 0.796)
Chang et al. [28]	1988–1997	Singapore	≤40, >40	55/583	Less HBV infection, worse liver function, less vascular invasion, earlier staged disease, but less surgically treated	Similar in all (*p*>0.05), but worse in early disease (*p* = 0.025)
Zhang et al. [29]	1988–2003	China (SEER)	≤45, >45	2102/25153	More males and higher tumor burden	Worse (*p*<0.001)
Poon et al. [14]	1989–1997	China	<70, ≥70	1116/222	More females, less HBV infected, more comorbidities, similar tumor stages, but less surgically treated	Similar (*p* = 0.94)
Lee et al. [10]	2003–2006	Korea	<65, ≥65	149/113	Less HBV infection, greater comorbidities, poorer performance status; similar tumor stages, but less surgically treated	Similar (*p* = 0.58)
Ozenne et al. [27]	2006–2008	France	<75, ≥75	337/43	More female, similar BCLC stages, less curatively treated	Similar (*p* = 0.74)
Borzio et al. [15]	2008–2015	Italy	≤70, >70	527/541	More females, HCV infected, better liver function, similar BCLC stages, less curatively treated	Similar (*p* = 0.586)
Ours	2008–2012	China	<65, ≥65	1212/318	More comorbidity, poorer performance status, more HCV infected, similar in BCLC stages 0-C, but less aggressively treated	Worse in all (*p* = 0.002), but similar after the same treatments

HBV, hepatitis B virus; HCV, hepatitis C virus; TACE, transarterial chemoembolization; SEER, Surveillance, Epidemiology, and End-Results; CLIP, the Cancer of the Liver Italian Programe; BCLC, Barcelona Clinics Liver Cancer

Our study identified that there were distinct differences of clinicopathological characteristics between younger and older patients. First, older patients with HCC were more likely to be HCV carriers, while younger patients were more HBV carriers. This finding may be explained that HBV is transmitted vertically in the perinatal period, whereas HCV is more infected at a later stage in life, and therefore patients with HBV-related HCC tended to be significantly younger than patients with HCV-related HCC [10, 14, 15, 21, 22]. Therefore, the average time at onset of HBV-related HCC was reported to be at least 10 years earlier than that of HCV-related HCC [23]. Interestingly, older patients had a higher proportion of non-HBV and non-HCV associated HCC, which implied that factors other than hepatitis virus, such as diabetes and accumulated genetic mutations, might be related to HCC development in some elderly patients [24]. Second, liver cirrhosis was less frequently presented in older than younger patients. These observations might be explained partially by the fact that hepatitis virus infection was less common in elderly patients. However, the liver function measured by Child-Pugh class seemed to be worse in elderly, indicating unsatisfactory control of liver disease. Last, more elderly patients presented with end-staged HCC (BCLC stage D) possibly due to less routine HCC screening and poorer performance status. However, the proportion of HCC within BCLC stages 0-A, B and C were similar distributed between older and younger patients. Consistently, some studies found similar tumor stages between older and younger patients with HCC [10, 14, 15, 25], but others reported earlier tumor stages in elderly patients [16, 21, 26] (Table 5). Thus, the discrepancy of tumor stages between older and younger patients must reflect the difference of patient inclusion and treatment indication of elderly patients in different institutions. In our experience, age is not a key factor in determining treatments. However, the function of vital organs and tolerance of treatments should be more meticulously evaluated in elderly patients before treatments.

Another important finding of the present study is that older patients had been less frequently treated with effective therapies than younger, which was consistent with some other studies [10, 14–16, 25–28] (Table 5). Elderly patients were likely to be more strictly selected for aggressive treatments probably due to relatively poor general condition, preexisting comorbidities and unwillingness to accept surgery [15, 22]. Surgical treatments were less performed in elderly patients across different BCLC stages. Although the percentage of patients undergoing curative treatments for BCLC 0-A staged HCC, including surgical resection, liver transplant and RFA in total, was equivalent between elderly and younger patients, it was notable that more elderly patients received RFA instead of surgical resection and liver transplant. Moreover, LRT were equally performed within BCLC B staged HCC between elderly and younger patients versus younger. Surgery or RFA, and TACE were recommended as first-line therapies for HCC of BCLC stage 0-A and B, respectively [29]. Therefore, it seemed that the same proportion of older and younger patients had been selected for first-line therapies according to the guidelines. However, surgery as a curative treatment was more frequently performed in intermediate and advanced stages of HCC in younger than older patients (BCLC stage B and C). Studies mostly from Asian countries have challenged BCLC treatment recommendations for its too strict restriction of surgical resection in stage B and C, and demonstrated improved survival by surgical resection over other palliative treatments in these patients [17–19]. But more elderly patients were treated only with BST across different BCLC stages. Therefore, it is likely that elderly patients have been less aggressively treated in most studies including ours not because of performance status or tumor burden, but subjective selection bias to treatments from both patient and doctor sides.

The results of the present study are limited by its retrospective design and single-center conduction, which needs further confirmation from more large prospective and multicenter studies. Although diabetes was mostly collect at admission, other co-morbidities were not routinely documented. However, ECOG score of each patient were assessed before surgery. Another major limitation in most previous studies and ours is that only inpatients were included during the time period [10, 14, 15, 25, 26]. However, as we believe, there were some outpatients missing mostly with old age and advanced disease but unadmitted and untreated. Therefore, studies based on national cancer registration dataset are needed to address the issue.

In conclusion, the present study supports equally effective treatments including surgery and LRT of HCC in elderly patients as selected. The survival of older patients was similar to that of younger patients with same tumor burden and after similar treatments. Future studies are needed to elucidate the selection criteria of the optimized treatments for older patients who are like “younger” and who are “older” physically. For example, ECOG score and tumor stages should be the critical considerations, rather than age simply, in selecting patients for appropriate treatment modalities.

## Supporting information

S1 File STROBE statement. (PDF)
